# Relevance of vertebral fractural deformity for hip fracture prediction: a community-based study of older Chinese women and men

**DOI:** 10.1007/s00256-025-05079-x

**Published:** 2025-11-17

**Authors:** Yì Xiáng J. Wáng, Jason C. S. Leung, Timothy C. Y. Kwok

**Affiliations:** 1 Department of Imaging and Interventional Radiology, Faculty of Medicine, The Chinese University of Hong Kong, Shatin, New Territories, Hong Kong SAR, China; 2 Jockey Club Centre for Osteoporosis Care and Control, Faculty of Medicine, The Chinese University of Hong Kong, Shatin, Hong Kong SAR, China; 3 Department of Medicine and Therapeutics, Faculty of Medicine, The Chinese University of Hong Kong, Shatin, New Territories, Hong Kong SAR, China

**Keywords:** Dual-energy X-ray absorptiometry, Bone mineral density, Vertebral fracture, Hip Fracture

## Abstract

**Objective:**

To estimate hip fracture (Fx) risk if one or more vertebral fractures are detected in an otherwise healthy older woman or man.

**Materials and methods:**

The study was conducted among older Chinese community subjects. 2000 women were enrolled; 1951 were followed for five years. 2000 men were enrolled; 1882 cases were followed for five years. With thoracolumbar spine radiographs, for each vertebra, a score of 0, -0.5, -1, -1.5, -2, -2.5, and -3 was assigned for no osteoporotic-like vertebral fractural deformity (OLVF) or OLVF of < 20%, ≥ 20 ~ 25%, ≥ 25% ~ 33%, ≥ 33% ~ 40%, ≥ 40% ~ 66%, and ≥ 66% vertebral height loss, respectively. OLVFss was the summed score of vertebrae T3 to L5. The femoral neck (FN) cutpoint T-score was ≤ -2.7 for osteoporosis in women and ≤ -2.1 for osteofrailia in men. OLVFss ≤  − 1.5 suggests osteoporosis in women, and OLVFss ≤  − 2.5 suggests osteofrailia in men.

**Results:**

For women, 26 cases developed hip Fx (mean age: 80 years). Baseline FN T-score ≤ -2.7 and OLVFss ≤ -1.5 had a positive predictive value of 5.22% and 5.05%, and a detection sensitivity of 65.38% and 53.85%, respectively. For men, 23 cases developed hip Fx (mean age: 81 years). Baseline FN T-score ≤ -2.1 and OVLFss ≤ -2.5 had a positive predictive value of 4.24% and 4.81%, and a detection sensitivity of 52.17% and 21.74%, respectively.

**Conclusion:**

Compared with FN T-score thresholds, OVLFss had a similar performance for future hip Fx risk estimation for women, for men OVLFss had a similar positive predictive value for future hip Fx risk but was associated with a lower detection sensitivity.

## Introduction

Osteoporosis is a systemic skeletal disease characterised by a reduction in bone mass and qualitative skeletal changes that cause an increase in bone fragility and a higher fracture risk. Assessment of vertebral fragility fracture (Fx) status, in addition to BMD (bone mineral density), aids in predicting fracture risk in older populations [1, 2]. The most relevant fracture site is the hip. In a Danish study published in 2010, compared with the general older population, the 1-year mortality after hip Fx increased by 27.2% in men and by 17.1% in women [3]. In a Hong Kong study published in 2016, mortality at 1 year after hip Fx was 27% in men and 15% in women [4]. In a Chinese study published in 2022, mortality at 1 year after hip Fx was 19.63% in men and 13.46% in women [5]. In addition to high mortality (the one-year mortality after hip Fx of around 20%), hip Fx patients are associated with poor functional recovery and often are unable to resume their pre-fracture function with a consequent deterioration in quality of life [3–6]. Because hip Fx typically necessitates hospitalization, data on their incidence is more reliable than data on other types of fractures. It is well documented that vertebral Fx shown on chest and abdominal CT examined are associated with increased further hip Fx risk. Buckens et al*.* [7] studied chest CT examinees of ≥ 40 years old and those later hospitalized for hip Fx during a median follow‐up of 4.4 years. After adjustment for age and gender, the presence of any vertebral Fx was associated with future hip fracture by a HRadj (adjusted Hazard Ratio) of 3.1. For men, the HRadj was 2.8, whereas it was 3.5 for women. Lee et al*.* [8] studied 204 hip Fx patients and 204 without fracture patients (controls). Hip Fx patients had abdominal CT performed within 6 years of the date that hip Fx occurred, 204 control subjects were from a screening CT colonography database. The mean interval from CT to hip Fx was 25.3 ± 20.8 months. The mean age of the case patients and control subjects was 74.3 ± 11.8 and 72.9 ± 10.65 years, respectively. In the cohort with future hip Fx, at least one prevalent moderate or severe VF was seen in 37 case patients who had a previous CT examination performed (18.1%), compared with five of 204 control subjects (2.5%). Skjødt et al. [9] identified CT scans including the chest and/or abdomen of 2000 consecutive men and women aged 50 years or older, and subjects were followed for up to 7 years. The risk of subsequent hip Fx for patients with vertebral Fx had HRadj of 3.02 [9]. These studies also have their limitations. The comparisons were conducted for patients with and without vertebral Fx, while it is likely that patients have a higher hip Fx risk compared to the general populations. Imaging data typically contained either chest CT or abdominal CT, thus not both thoracic and lumbar spine were assessed. Women’s fragility vertebral Fx and men’s fragility vertebral Fx should have different diagnostic criteria, with stricter threshold should being applied for men as mild and even moderate fractural deformity can be seen among men with normal bone strength [10–12], while these studies commonly use the same criteria for both men and women. Overall, these studies did not specifically answer such a question: if a vertebral Fx is detected in a screen subject, what would his/her hip Fx risk be in five years’ time?

While all data show the role of vertebral Fx as early manifestation of the deteriorated bone microarchitecture, what these vertebral Fx mean to an otherwise healthy subject from general community in terms of future hip Fx risk remains not well studied, particularly for East Asian populations. Based on the data from two population-based epidemiological studies conducted in Hong Kong for women and men, this study aims to answer two critical questions: 1) if one or more vertebral Fx of ‘sufficient severity’ are detected in an otherwise heathy older woman or man, what is the key message to this patient in term of further hip Fx risk? 2) what percentage of hip Fx occur in community subjects with vertebral Fx of ‘sufficient severity’? With hip Fx as the clinical endpoint [13], this study also compared a number of femoral neck (FN) and lumber spine T-score thresholds’ performance in prediction hip Fx risk for Chinese population. Following the 1994 WHO definition, densitometric osteoporosis prevalence among a population should be in proportion to its relative osteoporotic fracture risk with Caucasian female data as reference [14]. Earlier works had applied a standard T-score of −2.5 to define osteoporosis, recent works suggested that the cutpoint value for defining osteoporosis should be adjusted according to ethnic groups, gender, and the measured bone sites [15–18].

## Materials and methods

Osteoporotic fractures in women (MsOS) and in men (MrOS) Hong Kong studies represent the first large-scale prospective cohort studies conducted on bone health in East Asians. The studies were approved by the local institutional ethics committee. Written informed consent was obtained from all study participants. At baseline, 2000 Chinese men and 2000 Chinese women ≥ 65 years were recruited from the local communities from August 2001 to March 2003, to determine the relationship between anthropometric, lifestyle, medical, and other factors with BMD measured at the hip and spine. The subjects had a baseline mean age of 72.3 years (range, 65–92 years) for men and 72.5 years (range, 65–98 years) for women. The recruitment criteria were structured so that the study results would represent similarly aged community-dwelling ethnic Chinese men and women in Hong Kong. All subjects were able to walk without assistance, without bilateral hip replacement [19–22]. Men and women of similar age and from the same community-based population were investigated using the same methodology, thereby enabling a comparison of the results for men and for women. At baseline, BMD at the hip and lumbar spine (LS, L1–L4) was measured by Hologic QDR-4,500 W densitometers (Hologic, Inc., Waltham, Mass., USA). Local Hong Kong Chinese gender-specific reference data were used for the T-score calculation [23]. Left lateral thoracic and lumbar spine radiographs were obtained.

The four thousand men’s and women’s spine radiographs were evaluated for osteoporotic-like vertebral fractural deformity (OLVF). Vertebrae were evaluated with an extended version of semi-quantitative (eSQ) scheme with the following criteria [10, 24]: (1) minimal grade refers to radiological fractural deformity with < 20% height loss, which would be theoretically equivalent to Genant SQ grade 0.5, and the diagnosis of this grade rely on a distinct fracture-like change of a vertebra’s morphology (as compared with its expected shape considering those of the neighboring vertebrae); (2) mild grade fractural deformity is the same as Genant mild grade (≥ 20 ~ 25% height loss); (3) Genant moderate grade fractural deformity is divided into two subgrades: ≥ 25% ~ 33% height loss (moderate grade) and ≥ 33% ~ 40% height loss (moderately-severe grade); (4) Genant severe grade fractural deformity is divided into two subgrades: ≥ 40% ~ 66% height loss (severe grade) and with ≥ 66%height loss (collapsed grade). The vertebral height loss estimation was primarily based on measurement, by comparing the vertebral heights of neighboring vertebrae of normal shape, though it might not be necessary to take measurement for each OVLF for an experienced reader. Non-fractural changes, particularly osteoarthritic wedging and endplatitis short vertebrae, of the vertebrae shape were differentiated from OLVF as much as possible [10, 25–30]. Endplatitis short vertebrae refers to the acquired short vertebrae in which anterior and middle vertebral heights decreased to a similar extent but without apparent anterior wedging or bi-concave changes, and with at least two adjacent vertebrae involvement; endplate might show increased density but commonly without disc space narrowing (for diagnostic criteria see [30]). When a fractural deformity is judged to co-exist with other deformities, such an OLVF was also counted. The term ‘OLVF’ is used, as only based on spine radiograph it is not possible to absolutely diagnose a vertebral deformity as osteoporotic Fx in every case [23]. These spine radiograph data have been earlier read and reported [19–22]. For the current study, the radiographs were again read by a radiologist reader (YXJW) with > 10 years’ experience in reading spine fractural deformities, and consensus was reached with earlier readers.

For each vertebra in a subject, a score of 0 was assigned for no OLVF, −0.5 for OLVF of < 20% height loss, −1 for ≥ 20 ~ 25%, −1.5 for ≥ 25% ~ 33%, −2 for ≥ 33 ~ 40%, −2.5 for ≥ 40–66%, and −3 for ≥ 66% vertebral height loss, respectively. An OLVF sum score (OLVFss) was calculated by summing up the scores of vertebrae T3 to L5. Two adjacent minimal OLVF were assigned as −0.5, and three adjacent minimal OLVF were assigned to be −1. A minimal grade OLVF (score: −0.5) adjacent to a more severe grade OVLF (score: ≤ 1.0) was ignored for this minimal grade. For example, if T2 of −0.5 grade and L1 of −2.0 grade were seen, the total score for the T12 and L1 was only recorded as −2.0 (rather than −2.5). Earlier works have shown that, for Chinese and Italian women, OLVFss ≤  − 1.5 suggests the subject being osteoporotic; for Chinese men, OLVFss ≤  − 2.5 suggests the subject being osteofrailiac [11, 31–34].

There is strong evidence to suggest the cutpoint T-score value for defining osteoporosis among Chinese women should be ≤ −2.7 for femoral neck (FN) and ≤ −3.7 for lumbar spine, using Chinese women’s BMD reference values [18, 35–38]. For men, it has been noted that older men suffer from hip Fx at FN T-score approximately 0.5–0.6 higher than older women [33, 34, 39, 40]. A new category of low BMD status, osteofrailia, has been proposed, with femoral neck T-score ≤ −2.0 for older Caucasian men and femoral neck T-score ≤ −2.1 for older Chinese men [33, 34, 39]. While the FN osteoporosis classification fulfills the definition that osteoporosis prevalence is the same the hip Fx prevalence in older men, FN T-score ≤ −2.5 osteoporosis as a predictive diagnostic threshold misses the majority of hip Fx patients. On the other hand, FN T-score ≤ −2.0 for older Caucasian men and FN T-score ≤ −2.1 for older Chinese men osteoporosis as a predictive diagnostic threshold is expected to detect the majority of hip Fx patients [33, 39]. The osteofrailia T-score cutpoint value of lumbar spine among Chinese men has been suggested to be ≤ −2.5 [33, 34].

For the enrolled 2000 women, 50 died within the full four years’ follow-up. However, one of these subjects had one hip Fx at year-2 follow-up and later died of cancer, and was kept in this study. Thus, in total 1951 female cases were analysed. For the enrolled 2000 men, 121 of them died within the full 4 years’ follow-up. However, three cases of these subjects had hip Fx (one at year-1 follow-up, two at year-2 follow-up) during four years’ follow-up were kept in this study (causes of death: one with prostate cancer, one with obstructive pulmonary disease, and one with liver cirrhosis and liver uncompensated dysfunction). Thus, in total 1882 male cases were analysed.

Positive predictive value was the percentage hip Fx cases out of the total tested positive cases at baseline by a metric (for example, if 10 subjects were tested positive at baseline and among them 2 developed hip Fx during the follow-up, then the positive predictive value was 20%). Detection sensitivity was the percentage tested positive cases at baseline by a metric out of the total hip Fx cases (for example, if 10 subjects developed hip Fx during the follow-up, and among them 7 were initially tested positive at baseline, then the detection sensitivity was 70%). Other additional metrics, including lumbar spine T-score ≤ −2.5 for women, most severe OLVF being collapsed grade (i.e., OLVF score = −3.0) and most severe OLVF being severe grade (i.e., OLVF score = −2.5) for women and men, most severe OLVF being moderately-severe grade (i.e., OLVF score = −2.0) for men, OLVFss ≤ −2.0 and OLVFss ≤ −3.0 for men, were also tested for the purpose of comparison. The results for female FN T-score (≤ −2.7) were considered as the reference. Due to the limited sample size, data are presented descriptionally; no statistical analysis was conducted.

## Results

**Fig. 1:**
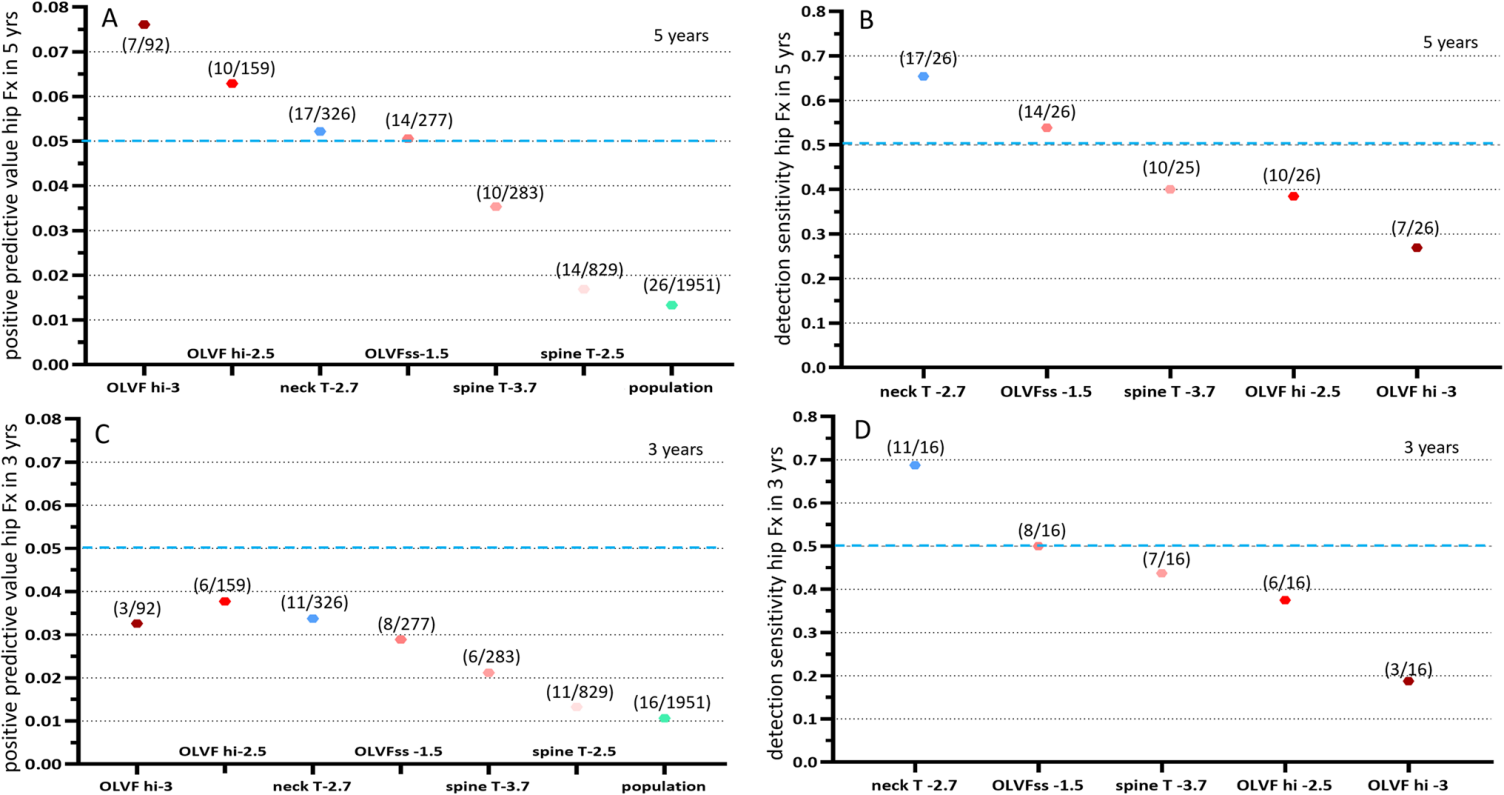
Women’s data, positive predictive value (**A**, **C**) and detection sensitivity (**B**, **D**) for hip Fx during five years’ (**A**, **B**) and three years’ follow-up (**C**, **D**). Femoral neck T-score ≤−2.7 (labeled ‘neck T-2.7’) is the reference metric. ‘Population’ denotes the hip Fx incidence among the study population during the follow-up. ‘Spine T −3.7’: lumbar spine T-score ≤−3.7; ‘spine T −2.5’: lumbar spine T-score ≤−2.5; ‘OLVF hi-3’: the most severe OLVF score being −3.0 (i.e., ≥66% vertebral height loss) for the study case; OLVF hi-2.5’: the most severe OLVF score being −2.5 (i.e., ≥40%vertebral height loss) for the study case; ‘OLVFss-1.5’: ‘OLVFss ≤−1.5’. (7/92) for ‘OLVF hi-3’ means: out of the 1951 subjects, 92 subjects had the most severe OLVF score being −3.0 at baseline, and of them 7 cases developed hip Fx during the follow-up. (17/26) for ‘neck T −2.7’ means: out of the 26 subjects who developed hip Fx during the follow-up, 17 of them had baseline femoral neck T-score ≤−2.7. In (B), one of the hip Fx cases missed lumbar spine T-score data. Blue dotted line: 5% threshold for positive predictive value and 50% threshold for detection sensitivity

For women, during the five years’ observation period, 26 cases had hip Fx (mean age at Fx: 80 years, range: 71–95 years), with an incidence of 1.333%. The positive predictive value was 7.609% for the threshold of the most severe OLVF score being −3.0, 6.289% for the most severe OLVF score being ≤ −2.5, 5.215% for FN T-score ≤ −2.7, 5.054% for OLVFss ≤ −1.5, 3.534% for lumbar spine T-score ≤ −3.7, and 1.688% for lumbar spine T-score ≤ −2.5, respectively (Fig. 1A). The hip Fx detection sensitivity was 65.38% for the threshold of FN T-score ≤ −2.7, 53.85% for OLVFss ≤ −1.5, 40.0% for lumbar spine T-score ≤ −3.7, 38.46% for the most severe OLVF score being ≤ −2.5, 26.92% for the most severe OLVF score being −3.0, respectively (Fig. 1B). For female subjects, during the three years’ observation period, 16 cases had hip Fx (mean: 79 years, range: 71–95 years), an incidence of 1.067%. The positive predictive value was all lower, being lower than 5% (Fig. 1C), while the detection sensitivity was similar to the five years’ results (Fig. 1D). The patterns of relative performance for each metric were broadly similar to the five years’ results.

**Fig. 2:**
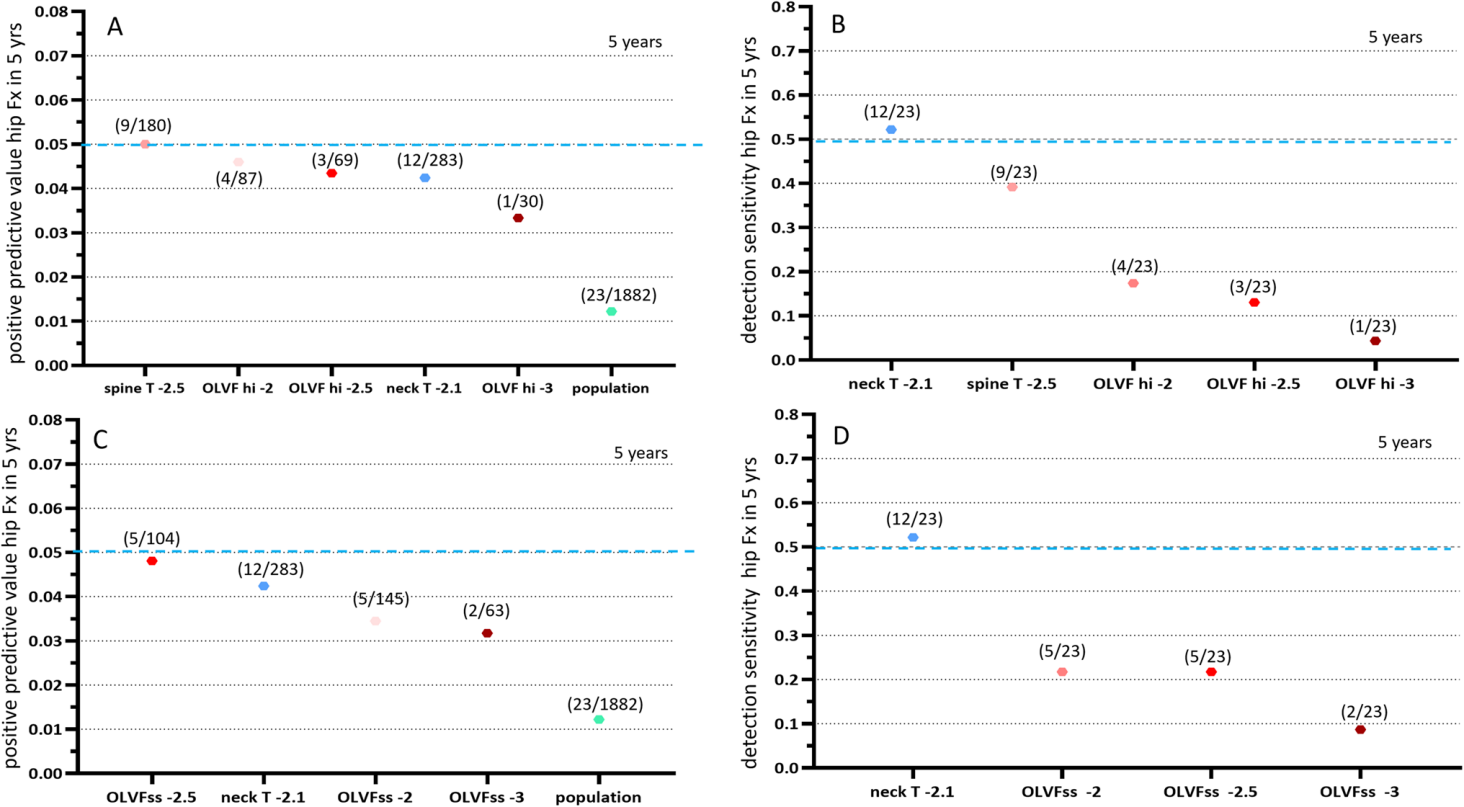
Men’s data, positive predictive value (**A**, **C**) and detection sensitivity (**B**, **D**) for hip Fx during five years’ follow-up. Femoral neck T-score ≤−2.1 (labeled ‘neck T-2.1’) is the reference metric. ‘Population’ denotes the hip Fx incidence among the study population during the follow-up. ‘spine T −2.5’: lumbar spine T-score ≤−2.5; ‘OLVF hi-3’: the most severe OLVF score being −3.0 (i.e., ≥ 66% vertebral height loss) for the study case; OLVF hi-2.5’: the most severe OLVF score being −2.5 (i.e., ≥ 40%vertebral height loss) for the study case; ‘OLVF hi-2.0’: the most severe OLVF score being −2.0 (i.e.,≥33% vertebral height loss) for the study case; ‘OLVFss-3’: ‘OLVFss ≤−3’; ‘OLVFss-2.5’: ‘OLVFss ≤−2.5’; OLVFss-2’: ‘OLVFss ≤−2’. (4/87) for ‘OLVF hi-2’ means: out of the 1882 subjects, 87 subjects had most severe OLVF score being −2.0, and of them 4 cases developed hip Fx during the follow-up. (12/23) for ‘neck T −2.1’ means: out of the 23 subjects who developed hip Fx during the follow-up, 12 of them had baseline femoral neck T-score ≤−2.1. Blue dotted line: 5% threshold for positive predictive value and 50% threshold for detection sensitivity

For male subjects, during the five years’ observation period, 23 cases had hip Fx (mean age at Fx: 81 years, range: 72–94 years), with an incidence of 1.222%. For metrics of the spine T-score ≤ −2.5, OLVFss ≤ 2.5, the most severe single OLVF score being ≤ −2.0, the most severe single OLVF score being ≤ −2.5, FN T-score ≤ −2.1, all had a positive predictive value between 5%−4% (Fig. 2). FN T-score ≤ −2.1 had a detection sensitivity of 52.17%, followed by lumbar spine T-score ≤ −2.5 of 39.13%. All OVLF metrics had a detection sensitivity of lower than 25% (Fig. 1B, D). Among OVLF metrics, OVLFss ≤ −2.5 had a positive predictive value of 4.808% and a detection sensitivity of 21.74%, and the most severe single OLVF score ≤ −2.0 had a positive predictive value of 4.598% and a detection sensitivity of 17.39%. These two performed better than other metrics (Fig. 2), and OVLFss ≤ −2.5 was slightly favored over the most severe single OLVF score ≤ −2.0. For men, during the three years’ observation period, 8 cases had hip Fx (mean age: 82 years, range: 76–92 years), with an incidence of 0.425%. Among these 8 cases, 6 cases (75%) had FN T-score ≤ −2.1 and 5 cases (62.5%) had lumbar spine T-score ≤ −2.5. Due to the small number of hip Fx cases available, analyses of positive predictive value and detection sensitivity for other metrics were not conducted.

## Discussion

Among all osteoporotic Fx, hip Fx incurs the greatest morbidity, mortality, and costs. This study further shows that, when an appropriate T-score cutpoint is applied, more than 70% of hip Fx occur among FN osteoporotic women (T-score ≤ −2.7) and osteofrailiac men (T-score ≤ −2.1). These results are consistent with our literature analyses [33], and highlight the value and importance of regular assessment of bone strength among older populations. For women, the OLVFss ≤ −1.5 criterion had a positive predictive value and detection sensitivity close to those of FN T-score criterion, while better than the lumbar spine T-score ≤−3.7 criterion. The most severe OLVF score ≤ −2.5 was associated with a higher positive predictive value than FN T-score ≤ −2.7, but with lower detection sensitivity (due to the lower prevalence of severe vertebral Fx). The criterion of the most severe OLVF being ≤ −2.5 had a similar detection sensitivity but higher positive predictive value compared with the criterion of lumbar spine T-score ≤ −3.7. These results suggest that, in scenarios where an FN BMD could not be measured, the OLVF assessment of spine can be used to replace FN BMD measure for women. As expected, the positive predictive value and detection sensitivity of FN BMD, lumbar spine BMD, and OLVF metrics of men’s results were overall slightly inferior to those for women [7, 22, 33]. FN T-score had a positive predictive value of 5.215% and detection sensitivity of 65.38% for women, and a positive predictive value of 4.240% and detection sensitivity of 52.17% for men. Among OVLF metrics, OVLFss ≤ −1.5 had a positive predictive value of 5.054% and a detection sensitivity of 53.85% for women; OVLFss ≤ −2.5 had a positive predictive value of 4.808% and a detection sensitivity of 21.74% for men. This could be at least partially due to the lower incidence of hip Fx and higher prevalence of non-osteoporotic vertebral deformity among men [12]. We have also reported that, in older Chinese men, OLVF at baseline barely predicts further vertebral radiographical Fx during four years’ follow-up [22].

A comparison of our OLVF results for hip Fx prediction with literature data will be difficult, as there were differences in study populations (different age, being healthy volunteers or patients), follow-up durations, and whether the whole thoracolumbar spine or only part of it was assessed. In our study, for women, OLVFss ≤ −1.5 had an increased hip Fx risk by a factor 3.79 (5.054/1.333) relative to general population; for men, OLVFss ≤ −2.5 had an increased hip Fx risk by a factor 3.93 (4.808/1.222) relative to general population. For the study reported by Buckens et al*.* with a median follow‐up of 4.4 years, HRadj (adjusted Hazard Ratio) was 2.8 for men and 3.5 for women. Skjødt et al. [9] studied older men and women with a follow up of 7 years, and risk of subsequent hip Fx had HRadj of 3.02 for patients with bassline vertebral Fx [9]. Our results show higher hip Fx risk ratios for subjects with vertebral Fx relative to the controls. This could be partially due to, among other factors, our controls were mostly healthy subjects and our OLVF assessment was more specific.

This study suggests the reasonableness of the cutpoint values applied in this study. That the OLVFss ≤ 1.5 criterion had a positive predictive value and detection sensitivity close to those of FN T-score osteoporosis threshold suggests the reasonableness of this OLVFss cutpoint value for women. On the other hand, with the women’s general population Fx incidence being 1.333%, lumbar spine T-score ≤ −2.5 showed a low positive predictive value being 1.688%. The criterion lumbar spine T-score ≤ −3.7, though still inferior to FN T-score, showed a better performance in predictive value than lumbar spine T-score ≤ −2.5. Lumbar osteofrailia spine T-score and OLVFss cutpoint value for Chinese men had not been well validated. It has been noted that OLVFss = −2 is not uncommon among younger men with normal bone strength [12], while OLVFss ≤ −2.5 or ≤ −3.0 are associated with features of fragility fracture [11, 34]. In this study, among OVLF metrics for men, OVLFss ≤ −2.5 performed best. The most severe single OLVF score ≤ −2.0, with a positive predictive value of 4.598% and a detection sensitivity of 17.39%, was the second best of OLVF metrics among men’s results. However, there is a concern that single vertebral deformity with 1/3 height loss (i.e., OLVF score = −2) could also be seen among subjects with normal bone strength [12]. This study confirms that lumbar spine T-score ≤ −2.5 is a reasonable compromise as the osteofrailia cutpoint for Chinese men. For the positive predictive value and detection sensitivity in men, lumbar spine T-score ≤ −2.5 performed close to the FN T-score and performed better than the OLVFss ≤ −2.5 criterion.

There are many limitations to this study. Though 2000 men and 2000 women were initially recruited from the community, the observed hip Fx numbers were still low, particularly for males’ three years observation period. Our results are considered preliminary and further validation should be conducted. Note that, the relevance of a positive predictive value is related to intervention regimens. For example, if a drug is effective, safe, and low cost, then a lower positive predictive value will be acceptable. During the observation period of the current study, 6 out of the 26 women with hip Fx in this study reported irregular anti-osteoporosis medication, while none of the men with hip Fx in this study reported anti-osteoporosis medication. Thus, our study was not a natural experiment without any intervention. However, it was known to us that regular medication was rare among our study participants during the five years follow-up observation. Yet another limitation is while we were able to register the hip Fx incidents during the observation period, the trauma energy levels were not always well defined. For subjects aged around 80 years, hip Fx due to high energy trauma are very uncommon [41, 42]. Finally, this is a single center study with only one ethnic group (i.e., Chinese). However, it is likely that our results, if further confirmed, will be likely applicable to other East Asian populations [43, 44]. Tentatively, it is also possible our results are relevant to Caucasian populations. Compared with East Asians, it is likely that Caucasian populations have proportionally higher OLVF prevalence/severity and higher hip Fx prevalence/incidence [18, 31, 37].

In conclusion, if vertebral Fx are detected in an otherwise healthy Chinese woman, OLVFss ≤ −1.5 predicts hip Fx risk of about 5.1% in five years’ time, and 53.9% of all hip Fx in five years’ time will occur in women with OLVFss ≤ −1.5. If vertebral Fx is detected in an otherwise healthy Chinese man, OLVFss ≤ −2.5 predicts hip Fx risk of about 4.8% in five years’ time, and 21.7% of all hip Fx in five years’ time will occur in men with OLVFss ≤ −2.5. For women, OVLFss evaluation has a similar performance for five years hip Fx risk estimation compared with the FN BMD osteoporosis threshold, and performs better than the lumbar spine BMD osteoporosis threshold. For men, OVLFss evaluation has a similar positive predictive value for future hip Fx risk as the FN BMD osteofrailia threshold, but is associated with a lower detection sensitivity.

## Data Availability

The data used in the current study are available from the corresponding author upon reasonable request.
